# Non-opioid Analgesics and the Endocannabinoid System

**DOI:** 10.4274/balkanmedj.galenos.2020.2020.6.66

**Published:** 2020-10-23

**Authors:** Ruhan Deniz Topuz, Özgür Gündüz, Çetin Hakan Karadağ, Ahmet Ulugöl

**Affiliations:** 1 Department of Medical Pharmacology, Trakya University School of Medicine, Edirne, Turkey

**Keywords:** Dipyrone, endocannabinoids, NSAIDs, paracetamol

## Abstract

Non-steroidal anti-inflammatory drugs produce antinociceptive effects mainly through peripheral cyclooxygenase inhibition. In opposition to the classical non-steroidal anti-inflammatory drugs, paracetamol and dipyrone exert weak anti-inflammatory activity, their antinociceptive effects appearing to be mostly due to mechanisms other than peripheral cyclooxygenase inhibition. In this review, we classify classical non-steroidal anti-inflammatory drugs, paracetamol and dipyrone as “non-opioid analgesics” and discuss the mechanisms mediating participation of the endocannabinoid system in their antinociceptive effects. Non-opioid analgesics and their metabolites may activate cannabinoid receptors, as well as elevate endocannabinoid levels through different mechanisms: reduction of endocannabinoid degradation via fatty acid amide hydrolase and/or cyclooxygenase-2 inhibition, mobilization of arachidonic acid for the biosynthesis of endocannabinoids due to cyclooxygenase inhibition, inhibition of endocannabinoid cellular uptake directly or through the inhibition of nitric oxide synthase production, and induction of endocannabinoid release.

Cannabinoids are a heterogenous group of compounds, which activate the cannabinoid receptors found throughout the body. They are not only found in the cannabis plant, but also produced by the human body (endocannabinoids). In addition, there are artificially synthetized synthetic cannabinoids, who are functionally similar to Δ9-tetrahydrocannabinol (THC), the main psychoactive and analgesic compound found in the plant (1,2). All of these compounds exert their effects via interaction with cannabinoid-1 (CB1) and cannabinoid-2 (CB2) receptors (3,4). Both CB1 and CB2 receptors are G-protein-coupled receptors, primarily exhibiting G_i/o_ signaling mechanisms. CB1 receptors are mainly expressed in brain structures, but also in peripheral tissues; on the other hand, CB2 receptors are expressed most abundantly in immune system cells in the periphery. Many of the unwanted effects of cannabinoid receptor agonists are caused via CB1 receptors located in the central nervous system (5,6).

Endocannabinoid system is comprised of CB1 and CB2 receptors, their endogenous lipid ligands (endocannabinoids) and the enzymes involved in their biosynthesis and inactivation. Endocannabinoids are derived from arachidonic acid (AA); anandamide [arachidonyl ethanolamide (AEA)] and 2-arachydonoyl-glycerol (2-AG) are best characterized and considered to be the main endocannabinoids (6,7). AEA and 2-AG are produced at post-synaptic neurons with two-step processes. Phosphatidylethanolamine is converted to N-acyl-phosphatidylethanolamine (NAPE) by the enzyme N-acyltransferase; then NAPE is hydrolyzed to N-acylethanolamines, such as AEA, by a NAPE-selective phospholipase D (NAPE-PLD). On the other hand, 2-AG is synthesized from diacylglycerol (DAG) by DAG lipase, following production of DAG from inositol phospholipids (7,8,9). Endocannabinoids are synthesized "on demand", released immediately, act in an autocrine or paracrine manner, and their biological actions rapidly terminate (8,10). AEA and 2-AG are removed from the extracellular space by a cellular uptake mechanism, followed by enzymatic inactivation. Relatively little is known about 2-AG uptake, but N-arachydonoyl-phenolamine (AM-404), the metabolite of paracetamol, is known to inhibit uptake of AEA. This is important with regard to the interaction of the analgesics and the endocannabinoid system, the main concept of this review. AEA is predominantly degraded to AA and ethanolamine by fatty acid amide hydrolase (FAAH), whereas 2-AG is predominantly metabolized to AA and glycerol by monoacylglycerol lipase (MAGL), and to a lesser extent by FAAH (9,11). In addition to FAAH and MAGL, AEA and 2-AG are demonstrated to be good substrates for cytochrome p450 monooxygenases, lipooxygenases and cyclooxygenases, mainly COX-2 (12,13,14). Besides FAAH and MAGL inhibition, these pathways of degradation are also important in elevation of endocannabinoid levels; since all non-steroidal anti-inflammatory drugs (NSAIDs) inhibit COX enzymes, COX-2 inhibition may participate in the antinociceptive effects of analgesic drugs.

Cannabis has been used for the management of pain for centuries, and numerous experimental and clinical research works have found effectiveness of the plant’s constituents, endocannabinoids and synthetic cannabinoids in different pain models (15,16,17). However, the number of approved cannabinoid-based medicines is small. Nausea and vomiting related to chemotherapy, anorexia related to AIDS, and chronic pain and spasticity associated with multiple sclerosis are the conditions that cannabinoids are approved for (6,18), but they are generally prescribed as an alternative and/or potential adjunctive agents in these indications. When targeting the endocannabinoid system as a promising future therapeutic strategy (19), inhibition of FAAH, MAGL and COX-2 seems to be one of the most attractive approaches (6,10,19,20). Such is due to the role of these enzymes in inactivation of endocannabinoids, with their inhibition increasing local endocannabinoid levels. The endocannabinoid system can be the target of many therapies, as it is involved in a number of physiological regulation pathways, but in this review we will focus mostly on modulation on nociception.

NSAIDs exhibit moderate analgesic, anti-inflammatory and antipyretic properties; they are the most common pain relief medicines in the world. The principal mechanism of action of NSAIDs is inhibiting the activity of COX enzymes, and thereby reducing the production of prostaglandins (21). However, accumulating evidence shows that NSAIDs’ therapeutic effects involve mechanisms other than COX inhibition, potentially including interaction with nitric oxide, opioidergic, monoaminergic and cholinergic systems (22). Involvement of the endocannabinoid system in the analgesic effects of NSAIDs also seems as a likely mechanism. Similar to cannabinoids/endocannabinoids (23,24), NSAIDs inhibit pain at the peripheral, spinal and supraspinal levels (25,26). Paracetamol and dipyrone are two different analgesic drugs, not being considered as classical NSAIDs, as they possess very little anti-inflammatory activity. However, the endocannabinoid system has been shown to participate in the antinociceptive actions of both NSAIDs, and paracetamol and dipyrone in recent years (27,28,29,30). In this review, we group classical NSAIDs, paracetamol and dipyrone together under the name of “non-opioid analgesics” and focus on the contribution of the endocannabinoid system for the antinociceptive effects of these analgesic drugs.

## Link between classical NSAIDs and the endocannabinoid system

Although the primary mechanism of action of NSAIDs is inhibition of COX enzymes, which are responsible for the production of prostaglandins, their ability to inhibit FAAH activity, responsible for the degradation of AEA, has also been shown. Augmenting endocannabinoid tonus locally, by inhibiting the degradative enzymes, may provide local efficacy in tissues, contributing for the control of nociception. In 1996, the potent anti-inflammatory drug indomethacin was suggested to reduce FAAH activity in the mouse uterus both *in vivo* and *in vitro* (31). Then, in a series of experiments, Fowler’s research group reported that several acidic NSAIDs, including ibuprofen, ketorolac, flurbiprofen, and some of their primary metabolites, inhibited FAAH (32,33,34). The inhibitory potency of these NSAIDs was relatively low, but increased 5-10-fold as the assay pH was reduced (35,36,37). These are very important findings, considering lowered pH in inflamed tissues together with effectiveness of local administrations and when acidic drugs are accumulated in these tissues. Accordingly, locally administered ibuprofen and rofecoxib produce synergistic effects with AEA, and this effect is blocked by a CB1 receptor antagonist (38,39). In a related study, indomethacin was shown to reduce carrageenan-induced edema, and a CB2 receptor antagonist was effective in preventing the NSAID’s action (40). In these studies, reduction of AEA metabolism via inhibition of FAAH activity is proposed as the mechanism of action for NSAIDs-induced antinociception; however, it should be taken into consideration that the inhibition of FAAH by NSAIDs does not appear to be potent (27,34,41).

Besides FAAH inhibition, another way of elevating endocannabinoid tonus via preventing their metabolism is COX-2 inhibition. The principal endocannabinoids AEA and 2-AG are good substrates for COX-2, producing prostaglandin-ethanolamides (prostamides) and prostaglandin-glycerol esters; a reduction in the levels of these proinflammatory and pronociceptive mediators may also contribute for their antinociceptive activity (12,13). There is an increasing interest on differential effects of NSAIDs on COX isoenzymes. Duggan et al. (42) indicated that (R) enantiomers of ibuprofen, naproxen and flurbiprofen are potent substrate-selective inhibitors of endocannabinoid oxygenation by COX-2; these NSAIDs are considered to be inactive as COX-2 inhibitors. Similarly, ibuprofen, mefamic acid and flurbiprofen are more potent inhibitors of COX-2-cyclooxygenation of 2-AG than of AA (42,43,44). Ibuprofen also exerts potent inhibition of AEA cyclooxygenation compared to AA oxygenation (41). Endocannabinoid-preferring COX inhibitors appear to be among potential novel analgesics; simultaneous FAAH and COX inhibition also seems to be an attractive target (27,45,46).

Increase in endocannabinoid tonus can be reached not only by decreasing their metabolism via inhibition of degradative enzymes, but also by augmenting endocannabinoid biosynthesis. Since AA is also important in endocannabinoid synthesis, COX inhibition probably provides more AA for endocannabinoid synthesis rather than prostaglandin synthesis (22,47). Indeed, it has been suggested that AA mobilization increases AEA production (48). Therefore, it seems that another mechanism implicated in the participation of endocannabinoids in NSAIDs’ effects is shunting of free AA from prostaglandin synthesis to endocannabinoid synthesis, although how AA participates in such production is not known.

Regarding the involvement of the endocannabinoid system in the analgesic effects of NSAIDs, Gühring et al. (49) proposed that, first, at the spinal level, indomethacin induces a shift of AA metabolism toward endocannabinoid synthesis; second, indomethacin lowers nitric oxide production, reducing activation of endocannabinoid transporters and thus breakdown of endocannabinoids; and third, it inhibits FAAH and hence enhances endocannabinoid levels. Spinal administration of flurbiprofen and intracerebroventricular administration of celecoxib also exerts endocannabinoid-dependent antinociception (50,51). Co-administration of ketorolac and the mixed CB1/CB2 cannabinoid receptor agonist WIN 55,212-2 produces an additive antinociceptive interaction in an inflammatory visceral pain model (16). Co-administration of a FAAH inhibitor and the COX inhibitor diclofenac also elicits a synergistic antinociceptive effect in the acetic acid model of visceral nociception (45). Contradictory findings are also worth mentioning; Silva et al. (52) reported that cannabinoid receptors do not seem to be involved in the peripheral antinociceptive mechanisms of dipyrone, diclofenac and indomethacin, following intra-plantar administration of the NSAIDs. Antagonism of cannabinoid receptors also does not influence diclofenac-induced antinociception when given systemically (53). In another study, neither the CB1 nor the CB2 antagonist blocked the effects of the NSAIDs in animals chronically administered with THC (54). Staniaszek et al. (55) concluded that nimesulide inhibits spinal neuronal responses in a CB1-dependent way, but they did not detect a concomitant elevation in AEA or 2-AG levels.

## Link between paracetamol and the endocannabinoid system

Paracetamol (acetaminophen) is one of the most widely used drugs as an antipyretic and analgesic. Unlike classical NSAIDs, paracetamol does not exert any anti-inflammatory activity, whereas its analgesic activity is similar to that of NSAIDs. Inhibition of peripheral COX enzymes does not appear to be primarily responsible for the antinociceptive activity of paracetamol; but probably some central mechanisms, including the endocannabinoid system, participate in these effects (56). Inhibition of central COX, modulation of serotonergic and opioidergic systems, and inhibition of nitric oxide synthetases (NOS) are among the proposed mechanisms (57,58,59,60). In 2005, Högestätt et al. (61) reported that paracetamol, following deacetylation to p-aminophenol, is FAAH-dependently conjugated with AA in the brain and spinal cord to form the bioactive AM-404. Then, CB1 receptors have been demonstrated to participate in both local and systemic antinociceptive effects of paracetamol (62,63). In their detailed research, Mallet et al. (28) suggested that AM-404 indirectly activates the supraspinal CB1 receptors, which in turn reinforces the activity of descending serotonergic inhibitory pathways. The metabolite AM-404 was already known to have the ability of inhibiting uptake of AEA; moreover, it has also shown to be a central COX inhibitor, a FAAH inhibitor, a weak CB1 activator, and a potent activator of TRPV1 (46,61,64,65,66,67). All of these properties of AM-404 may be related to its mediatory role in the antinociceptive activity of paracetamol. Results of another study implied that modulation of the endocannabinoid system mediates the synergistic antinociceptive effects of paracetamol combinations (68). There are also some contrary data, indicating that cannabinoid receptor antagonists do not block the effects of paracetamol, but these results were obtained in animals following chronic administration of THC or in an acute visceral pain model (54,69).

In most of these studies, pharmacological blockade or genetic deletion of cannabinoid receptors have been performed. Our group measured local endocannabinoid and N-acylethanolamide levels in the brain and spinal cord of rats, in order to observe the interaction of paracetamol and endocannabinoids directly; we observed an increase in 2-AG levels in the PAG and the RVM 12 h after paracetamol administration, but a decrease in AEA levels in the RVM and spinal cord (70). There are also studies on the contribution of the endocannabinoid system to some other effects of paracetamol. It was suggested that paracetamol exhibits a dose-dependent anxiolytic effect in mice via cannabinoid CB1 receptors (71). Paracetamol was also shown to enhance social behavior and cortical cannabinoid levels in mice in a CB1-mediated way (72). On the other hand, antagonism of cannabinoid CB1 and CB2 receptors does not prevent the antipruritic effect of systemic paracetamol (73).

## Link between dipyrone and the endocannabinoid system

Dipyrone (metamizole) is another worldwide used antipyretic and analgesic drug. Unlike classical NSAIDs, but similar to paracetamol, it possesses little anti-inflammatory activity. Despite intensive research, the precise mechanism underlying the antinociceptive effect of dipyrone is still unknown. Rather than peripheral COX inhibition, it has been suspected for a long time that dipyrone elicits centrally-mediated antinociceptive action (74,75,76). Initially, research has focused on the concept that endogenous opioids are involved in dipyrone’s antinociception. When microinjected into the PAG, dipyrone exerts antinociceptive effects mediated by endogenous opioids of the RVM (77), which then triggers descending inhibition of spinal nociception (78). The role of endogenous opioids in the spinal cord was also demonstrated (79). In another study, PAG-administered dipyrone induced development of tolerance in rats (80). When administered intravenously, dipyrone also causes anti-nociception by activating the endogenous opioid system (81). Other than its interaction with endogenous opioids, dipyrone is suggested to possess (weak) antinociceptive activity by classical COX inhibition (82), and by activation of the L-arginine-nitric oxide pathway and subsequent K_ATP_ channel opening (83), although there are some opposite findings (84,85).

In 2012, Rogosch et al. (29) demonstrated that two unknown metabolites of dipyrone form in the brain and spinal cord. FAAH seems to be responsible for the formation of these metabolites, and once formed, they bind weakly to cannabinoid receptors, but are modest inhibitors of COX-1 and -2. Then, it was shown that microinjection of dipyrone into the PAG elicits antinociception via CB1 receptors in an inflammatory pain model (86). There are studies that suggest mechanisms other than the endocannabinoid system for the antinociceptive effects of dipyrone, but these results were obtained under non-inflammatory conditions (30,87). However, the majority of the reports point to the important role of the endocannabinoid system in antinociception induced by dipyrone. It is suggested that activation of CB1, but not CB2 receptors, together with neuronal K_ATP_ opening is involved in the antihyperalgesic effect of dipyrone metabolites (88). These novel metabolites reduce the activity of ON-cells and enhance the activity of OFF-cells in the RVM (25). Importantly, Crunfli et al. (47) indicated that the endocannabinoid system, especially CB1 receptors, is involved in analgesia, catalepsy and hypolocomotion induced by systemic dipyrone. They hypothesized that COX and FAAH inhibition together may increase endocannabinoid availability and exhibit the above-mentioned effects via CB1 receptor stimulation. In accordance with these reports, a computational analysis suggested dipyrone metabolite 4-methylaminoantipyrine as a CB1 receptor agonist (89). In the research mentioned in the paracetamol section, we also measured local endocannabinoid levels in the brain and spinal cord of rats following systemic dipyrone administration; dipyrone exerts no action on 2-AG levels, but unexpectedly leads to a reduction in AEA levels in the RVM and spinal cord (70). In a very recent study, dipyrone, following hydrolysis to its active metabolite 4-methylaminoantipyrine, exerted a local antihyperalgesic effect partially dependent on CB2 and kappa-opioid receptors (90). Regarding studies that focus on domains beyond nociception, unlike paracetamol, systemic dipyrone does not exert anxiolytic-like effects in mice (91).

Cannabinoids modulate nociception at the peripheral, spinal and supraspinal levels (23,24). After activating supraspinal cannabinoid receptors, cannabinoids inhibit the presynaptic release of GABA via CB1 receptors in the lateral-ventrolateral PAG and RVM, and hence increase the postsynaptic neuron activity (92,93,94,95). In addition to their peripheral actions, non-opioid analgesics and/or their metabolites may augment endocannabinoid levels and/or directly activate cannabinoid receptors, facilitating the activity of descending inhibitory pathways, and thus decreasing nociceptive transmission.

**Figure 1:**
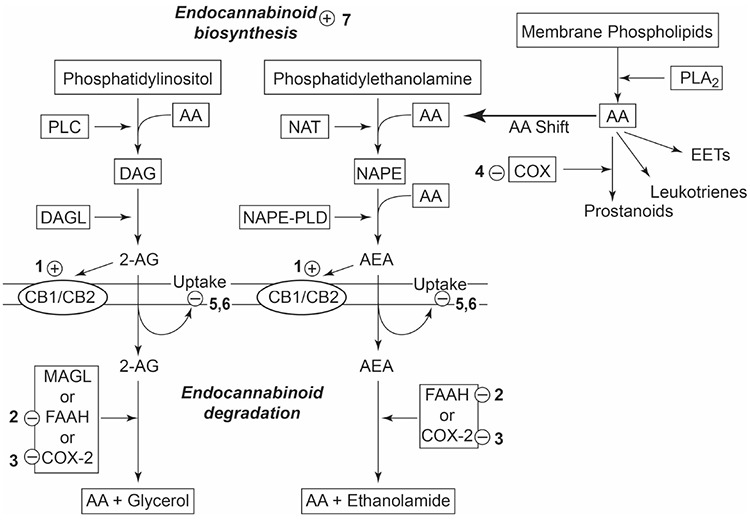
Possible mechanisms of action regarding to contribution of the endocannabinoid system to the antinociceptive effects of non-opioid analgesics. Non-opioid analgesic drugs and their metabolites; 1) may activate cannabinoid receptors, 2, 3) may reduce endocannabinoid degradation via FAAH and/or COX-2 inhibition, 4) may induce arachidonic acid shift to endocannabinoid biosynthesis, 5, 6) may inhibit cellular uptake directly or via inhibiting nitric oxide synthase production, and finally 7) may stimulate endocannabinoid release.

We conclude that the endocannabinoid system may participate in the antinociceptive effects of non-opioid analgesics via several mechanisms (Figure 1):

1- Activation of cannabinoid CB1 receptors (peripheral, spinal, supraspinal) by non-opioid analgesics and/or their metabolites (29,88,90);

2- Increase in endocannabinoid levels by;

a) inhibition of degradative enzymes;

 i) via FAAH inhibition (34,41);

 ii) via COX-2 inhibition (27,42,49);

b) shifting AA metabolism toward endocannabinoid synthesis due to COX inhibition (47,48,49);

c) reducing activation of endocannabinoid transporters and thus endocannabinoid degradation due to inhibition of NOS production (96,97);

d) induction of endocannabinoid release (70,94);

e) inhibition of cellular uptake of endocannabinoids by the metabolite (paracetamol) (22,61).
